# Three-dimensional cellular automaton simulation of coupled hydrogen porosity and microstructure during solidification of ternary aluminum alloys

**DOI:** 10.1038/s41598-019-49531-0

**Published:** 2019-09-11

**Authors:** Cheng Gu, Yan Lu, Colin D. Ridgeway, Emre Cinkilic, Alan A. Luo

**Affiliations:** 1 Department of Materials Science and Engineering, The Ohio State University, Columbus, OH 43210 USA; 2 Department of Integrated Systems Engineering, The Ohio State University, Columbus, OH 43210 USA

**Keywords:** Metals and alloys, Computational science

## Abstract

Hydrogen-induced porosity formed during solidification of aluminum-based alloys has been a major issue adversely affecting the performance of solidification products such as castings, welds or additively manufactured components. A three-dimensional cellular automaton model was developed, for the first time, to predict the formation and evolution of hydrogen porosity coupled with grain growth during solidification of a ternary Al-7wt.%Si-0.3wt.%Mg alloy. The simulation results fully describe the concurrent nucleation and evolution of both alloy grains and hydrogen porosity, yielding the morphology of multiple grains as well as the porosity size and distribution. This model, successfully validated by X-ray micro-tomographic measurements and optical microscopy of a wedge die casting, provides a critical tool for minimizing/controlling porosity formation in solidification products.

## Introduction

Aluminum alloys are widely used in automobile, aerospace, and other industrial applications. Aside from the advantages of high strength-to-weight ratio, good corrosion resistance, and low cost, porosity exists ubiquitously in aluminum solidification products. Porosity formed during solidification has been a major issue adversely affecting the performance, in particular the ultimate strength and fatigue resistance, of solidification products such as castings, welds or additively manufactured components. There are two major types of porosity observed in solidification products^1,2^: (1) shrinkage porosity due to solidification shrinkage and inadequate feeding during processing, and (2) gas porosity due to air entrapment and insoluble gases such as hydrogen. Hydrogen porosity, caused by the large difference in the solubility of hydrogen between liquid and solid aluminum phases, is a major source of porosity in aluminum solidification products. Therefore, the present research focuses on the porosity developed by the presence of atomic hydrogen dissolved in molten aluminum.

In order to improve the mechanical properties of final products, many researchers have experimented on the gas porosity and hydrogen diffusion during casting and welding processes of aluminum alloys^3–8^. Haboudou *et al*.^4^ carried out laser welding experiments of A356 and AA5038 alloys, where they found that a surface preparation (especially laser cleaning) could reduce the hydrogen porosity in the weld bead. Weingarten *et al*.^8^ found that hydrogen porosity can be reduced by efficient drying of the powder in selective laser melting experiment of AlSi10Mg alloy. Dispinar *et al*.^9,10^ conducted several casting experiments of A356 alloy with different pre-treatments to obtain various hydrogen levels. Additionally, Kaufmann and Rooy^2^ showed that hydrogen porosity could be influenced by the cooling rate of aluminum casting. Felberbaum *et al*.^7^ performed *in situ* X-ray tomography on Al-Cu alloys, and calculated an effective hydrogen diffusion coefficient as a function of the volume fraction of solid. These studies suggest that reducing the hydrogen source or increasing the speed of pores moving out of melt is beneficial to reducing the total porosity and the final properties of solidification products. Although experiments can be conducted to measure porosity directly after the products are made, it is difficult to understand the nucleation and evolution of porosity during solidification and predict the porosity formation in the final products for product design and process optimization. Modeling and prediction of porosity formation has become a major goal in modeling of solidification microstructure, which provides an essential link in integrated computational materials engineering^11–13^ (ICME) for solidification products.

Several studies on analytical solutions and numerical simulation methods have been carried out to understand the mechanisms of nucleation and evolution of hydrogen porosity during solidification. Li and Chang^14^ described an analytical solution for nucleation and growth of hydrogen pores during solidification of A356 alloy. This analytical method calculates the temperature range of nucleation, the fraction of solid at nucleation, and the supersaturation of hydrogen needed for nucleation. Lee *et al*.^1^ reviewed both analytical and numerical modeling approaches for micro-porosity formation in aluminum alloys. This study provided useful insights into the new models which govern the pore formation and its interaction with evolving grain structure. Following their initial work, Lee and co-authors carried out a series of studies on the porosity formation including mathematical modeling^15^, *in situ* observation^3,16^, and cellular automaton (CA) simulation^17,18^. Later, Tiedje *et al*.^19^ investigated the effect of cooling conditions on porosity formation in Al-Si alloys, and proposed a multi-zone model to explain the porosity in various regions of a casting. However, the nucleation and evolution of porosity during solidification were not clearly presented in the study^19^. Meidani *et al*.^20,21^ developed two-dimensional (2-D) and three-dimensional (3-D) multiphase-field models to study the formation and growth of a micropore constrained in a solid network. Unfortunately, their work only focused on the morphological analysis and pinching effect, and neither the porosity size nor the porosity distribution was considered. Karagadde *et al*.^22,23^ proposed a level-set method to simulate the hydrogen bubble evolution and engulfment during solidification, and then extended the study from 2-D to 3-D^24^. However, the surrounding solidified microstructure was not considered, and coupling interaction between dendrite and porosity could not be described in their model. Jie *et al*.^25,26^ adopted a mathematical model on the microporosity of aluminum castings, incorporating the pressure increase induced by dissolved hydrogen, solidification shrinkage and interface energy. Nevertheless, the model could not provide the graphical morphology output of the subject porosity. Zhu *et al*.^27^ proposed a 2-D CA-FDM model to simulate the growth of microporosity and dendrite during solidification of Al-Si alloys, but was not able to extend the model to the third dimension. Du *et al*.^28^ adopted phase field method to study gas bubble nucleation and growth with the microstructure evolution during solidification of pure aluminum. However, the simulation was performed in 2-D and a small calculation domain. Most of the related research has focused on predicting the final porosity level in a solidified casting of binary alloys, while modeling and simulation of porosity size and porosity distribution with graphical morphology output are very limited. It is necessary and critical to predict porosity coupled with dendrite growth of multi-component alloys in an ICME framework to obtain location-specific microstructure models for location-specific mechanical property predictions^29^. By doing so, the mechanism of formation and morphological changes of both dendrite and porosity can be understood and used to reduce porosity in solidification products at the location specific level, which serves an essential part in ICME for the manufacturing industry.

**Figure 1:**
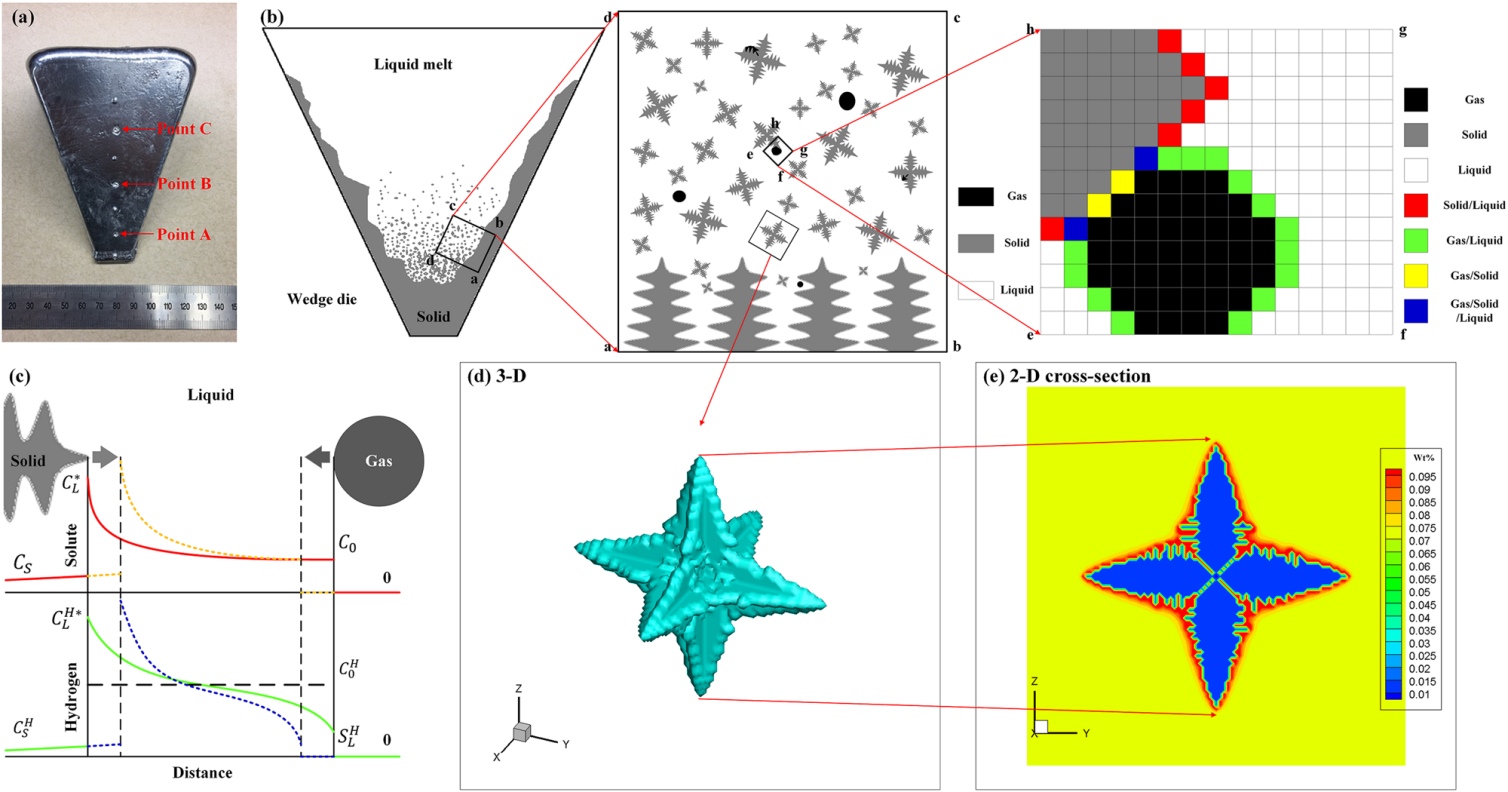
Schematic diagram of cellular automaton simulation (**a**) wedge casting sample, (**b**) illustration of multi-scale grains and porosity during solidification, (**c**) solute and hydrogen distribution in the liquid between solid and gas (red solid line: solute distribution at earlier stage; yellow dashed line: solute distribution at later stage; green solid line: hydrogen distribution at earlier stage; blue dashed line: hydrogen distribution at later stage), (**d**) 3-D single dendrite, and (**e**) 2-D single dendrite.

In this paper, a 3-D CA model was developed to simulate the formation and evolution of hydrogen porosity during solidification of an Al-7wt.%Si-0.3wt.%Mg ternary alloy (schematic diagram is shown in Fig. 1). Grain growth was considered in the model, and porosity evolution with growth of multi-grains over time was simulated as well. Solute and hydrogen distributions were obtained based on diffusion during solidification. Porosity morphology was then visualized from the simulated results, and the porosity size and distribution were computed. The effects of initial hydrogen concentrations and cooling rates on porosity evolution were investigated. Finally, the simulation results were validated by comparing with the experimental results from a wedge die casting.

## Results and Discussion

**Figure 2:**
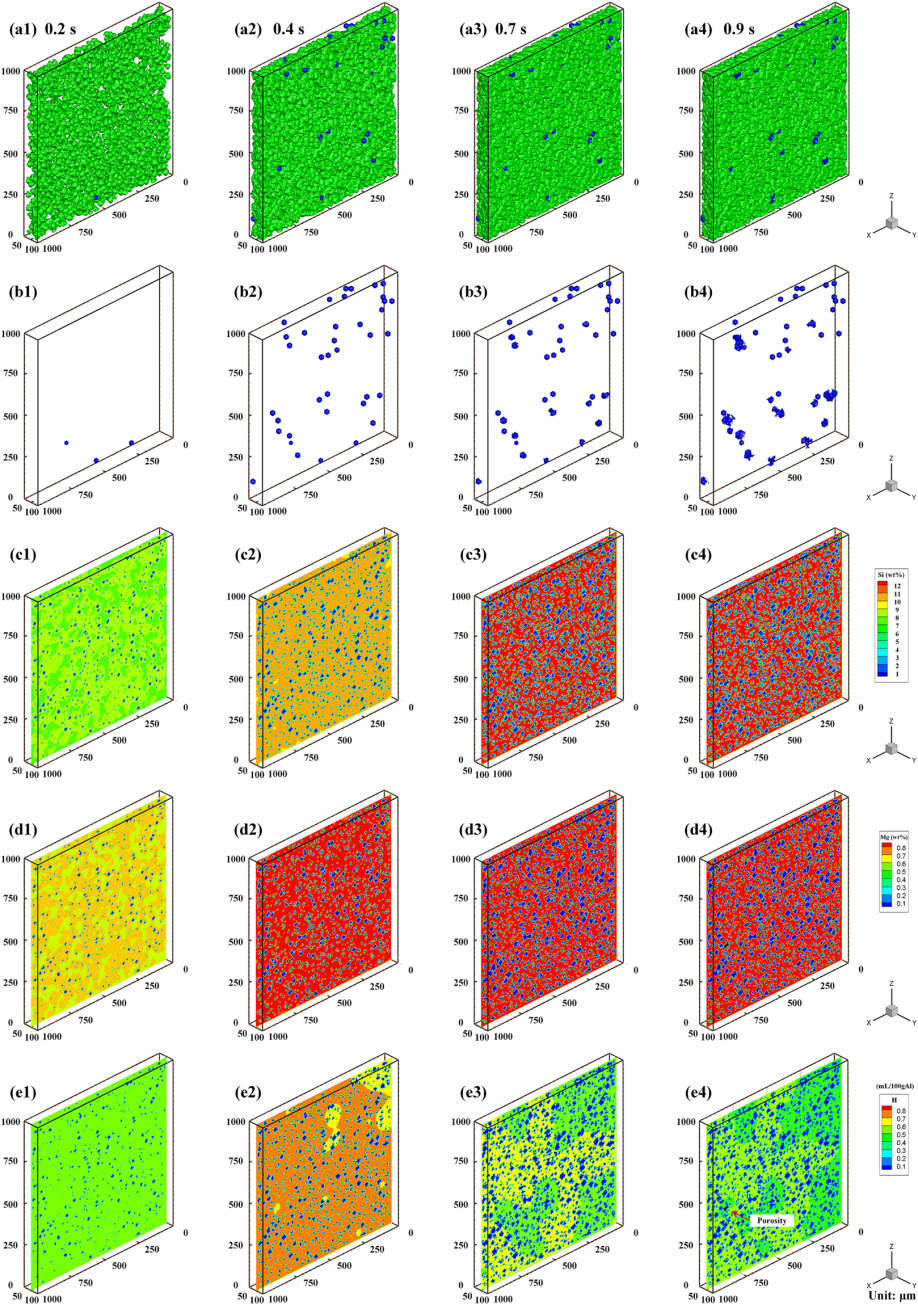
Evolutions of (**a**) grain, **(b**) porosity, (**c**) solute (Si) concentration field, (**d**) solute (Mg) concentration field, and (**e**) hydrogen concentration field with a cooling rate of 50 K/s and an initial hydrogen concentration of 0.5 mL/100 g Al at different times and temperatures: (**a1,b1,c1,d1,e1**) 0.2 s, 886 K (613 °C); (**a2,b2,c2,d2,e2**) 0.4 s, 876 K (603 °C); (**a3,b3,c3,d3,e3**) 0.7 s, 861 K (588 °C); (**a4,b4,c4,d4,e4**) 0.9 s, 851 K (578 °C). (Grains are shown in green in (**a**), and blue in (**c**,**d**,**e**); porosity is shown in blue in (**a**,**b**), and black in (**c**,**d**,**e)**).

Grain nucleation/growth as well as porosity nucleation/growth were simulated using the CA model established in this paper. The calculation domain is comprised of a 200 × 200 × 20 mesh cube with a uniform cubic mesh size 5 μm. Symmetrical boundary conditions are used on all the six surfaces of the calculation domain. The temperature is assumed to be homogeneous in the entire domain with a constant cooling rate 50 K/s, and the initial temperature is the liquidus temperature. The porosity nucleation parameters used are as follows: the initial hydrogen concentration in the melt is set as 0.5 mL/100 g Al, the maximum pore nucleation density ${N}_{H}^{max}=1\times {10}^{11}$ m^−3^, the maximum and minimum pore nucleation saturations ${S}_{H}^{max}=2.0$ and ${S}_{H}^{min}=1.4$, and the critical saturation criterion ${S}_{H}^{N}=1.2$. As solidification proceeds, the melt temperature decreases, nucleation and growth of grains occurs, and hydrogen pores start to form and grow within the calculation domain. Figure 2 shows simulated results of both grains and porosity at different time steps, allowing the examination of the grain and porosity morphologies, solute concentrations (Si and Mg), as well as hydrogen concentrations at different time steps.

It can be seen from Fig. 2(a1–a4,b1–b4) that the growth of grains and porosity are related and their structures are intertwined. The formation of porosity hinders the growth of grains, and vice versa. As the gas pores are surrounded by solidifying grains, they become enclosed and are unable to escape from the liquid, resulting in porosity. Figure 2(c1–c4,d1–d4,e1–e4) show that the hydrogen distribution is significantly different from the Si and Mg distributions. As the temperature decreases, the solute concentration in the remaining liquid is always found to increase due to solute rejection from solid to liquid during liquid-solid transformation. Furthermore, the nucleation and growth of pores do not affect the distributions or the diffusion coefficients of Si and Mg in Al. When the temperature reaches the eutectic temperature, the Si concentration in the remaining liquid increases to the eutectic concentration of 12.6 wt.% as shown in Fig. 2(c4). Conversely, the hydrogen concentration first increases and then decreases in the latter stages of solidification. During the liquid-solid transformation the temperature of the melt will decrease and is accompanied by grain growth and an initial increase in the hydrogen concentration. However, when the hydrogen concentration in the liquid is increased to a value larger than the hydrogen saturation limit and the nucleation criterion is fulfilled, the nucleation of hydrogen gas pore occurs. Stable hydrogen pores are then able to grow and act as sinks where hydrogen is absorbed within the pore, resulting in a decreased hydrogen concentration in the remaining liquid. In addition, the hydrogen diffusivity is much larger than solute diffusivity, which results in a hydrogen concentration that is less accumulated in the liquid near S/L interface compared to the Si and Mg concentrations as shown in Fig. 2(c1,d1,e1).

**Figure 3:**
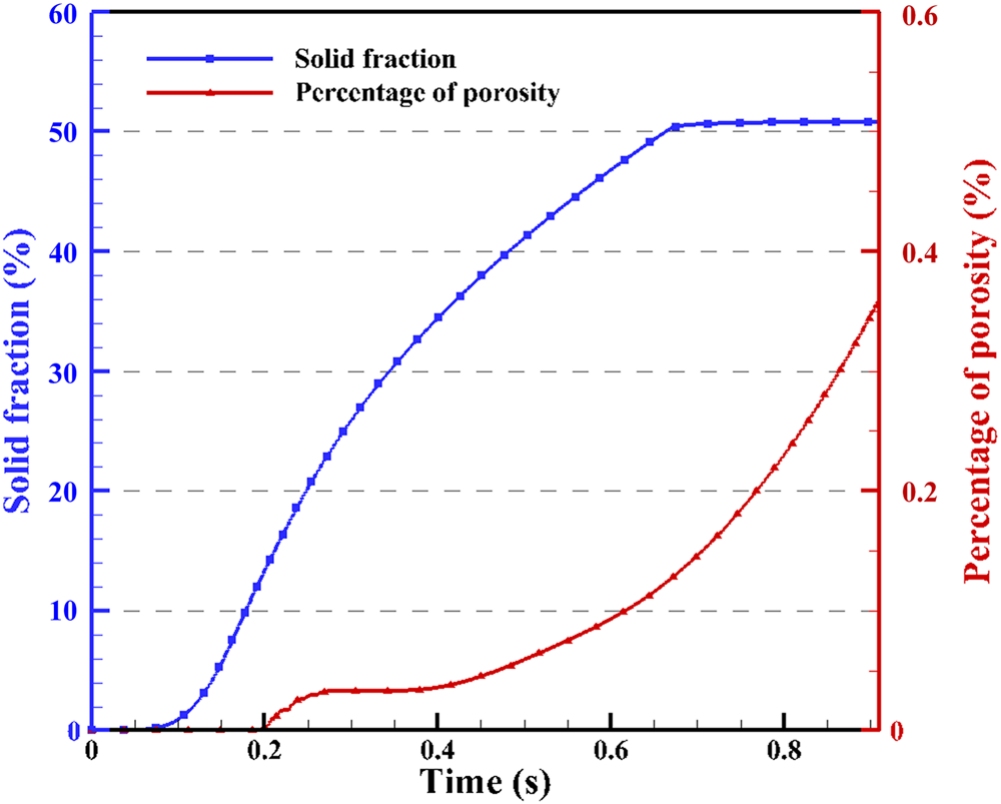
Solid fraction and porosity percentage vs. time during solidification.

The corresponding solid fraction and porosity percentage with time were recorded quantitatively as shown in Fig. 3. During the solidification process, Fig. 3 shows that the solid fraction of primary Al increases first, and then converges to a constant value. It can be seen that the solid fraction increases after the beginning of solidification, and the increasing rate of the solid fraction increases from zero, and then decreases to zero. This phenomenon can be described by the nucleation and growth of grains and the effects of undercooling and cooling rate. Since the existence of Si particles, Mg_2_Si, and eutectic solidification were ignored in the simulation, the solid fraction of primary Al could not increase when the Si concentration in the remaining liquid reaches eutectic concentration of Si (12.6 wt.% in this case) and all remaining liquid is considered as eutectic. Such an assumption explains in the flat stage of the solid fraction that appears at the end of solidification.

Also as shown in Fig. 3, the percentage of porosity increases from zero to a constant value first, and is followed by an exponential increase. The solid fraction increases earlier than the percentage of porosity, which is expected due to the larger hydrogen solubility limit within the liquid. In the initial stage of the solidification, the porosity percentage remains zero, which means there is an incubation time for porosity nucleation. As solidification continues and the liquid-solid transformation increases the hydrogen concentration in the liquid, the nucleation of the pores will occur as long as the local hydrogen concentration is larger than the local hydrogen saturation and the nucleation criterion is satisfied. It should be noted that after the pore nucleation, the hydrogen solubility changes greatly. The small initial radius of the nucleated pore leads to a large internal pressure, and results in a large hydrogen solubility. On the other hand, the nucleation of the pores will absorb hydrogen around them. Thus, pores are not able to grow immediately after nucleation as the local hydrogen concentration is lower than the local hydrogen solubility. The incubation time which appears in pore nucleation and in pore growth has been previously reported^27^. As solidification proceeds, the local hydrogen concentration in the melt continues increasing. When the local hydrogen concentration near the pore is higher than local hydrogen solubility limit again, the pore will start to grow, resulting in higher percentage of porosity. It can be seen that the porosity percentage rapidly increases, and the initially increased rate of solid fraction slows down as the temperature is lower than 870 K (597 °C).

**Figure 4:**
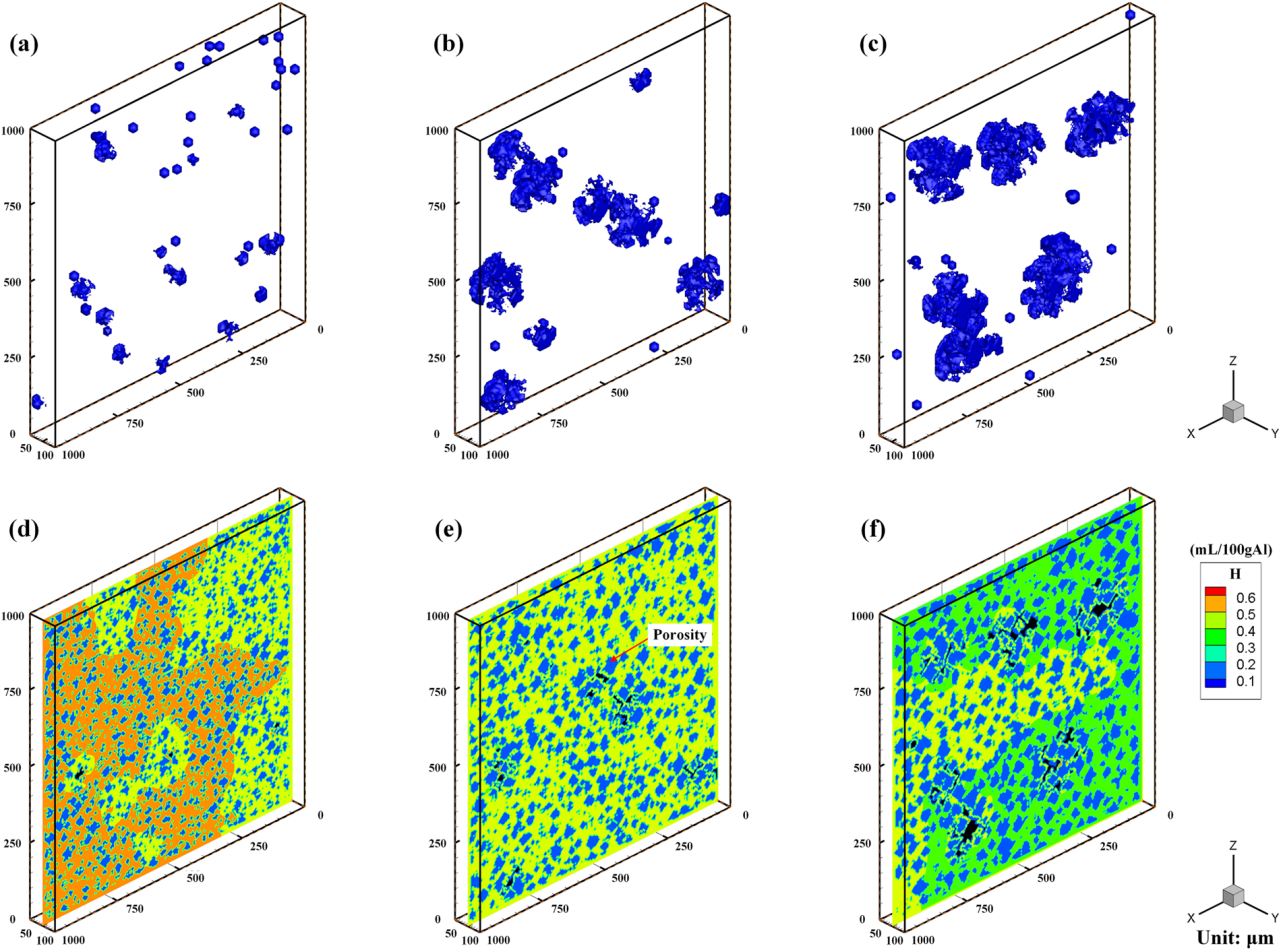
Simulated porosity and hydrogen concentration field with different cooling rates: (**a,d**) 50 K/s; (**b,e**) 10 K/s; and (**c,f**) 5 K/s.

Figure 4 shows the simulated morphologies of porosity and hydrogen concentration fields when the temperature is decreased to the eutectic temperature point with various cooling rates of 50 K/s, 10 K/s and 5 K/s. It can be clearly observed that as the cooling rate is decreased, the total number of individual pores decreases, yet the size of individual pores increases. Similarly, the lower cooling rate is also shown to increase the grain size as shown in Fig. 4(d,e). Conversely, it is known that a higher cooling rate results in increased nucleation of grains, faster grain growth velocity, and thus a finer grain size. The increased number of solidified grains hinders the diffusion of hydrogen in the remaining liquid and the growth of the nucleated pores. Due to the effect of the interfacial tension at Gas/Liquid interface, the nucleated pores should grow spherically. With the growth of grains and pores, the growth of pores is blocked, leading to irregular shapes of the pores. The hindered growth leads to an increase in the total number of individual pores as the melt has less time for hydrogen diffusion and pore growth, thus, smaller size and increased number of porosity. Less time for hydrogen diffusion also has a consequence on the hydrogen concentration in the remaining liquid, as more hydrogen will be present in the remaining liquid compared to lower cooling rates. The larger the pore radius, which is associated with lower cooling rates, the lower the local hydrogen saturation. Therefore, more hydrogen molecules will contribute to the growth of pores, which results in the lower hydrogen concentration within the melt at the end of solidification. On the other hand, several pores have large radii, while the other pores are very small as shown in Fig. 4(c). It should be noted that the internal pressure *P*_*G*_ of a large pore is lower than that of a small pore, which leads to a lower solubility of a large pore than that of a small pore. The lower the internal pressure and solubility, the larger the gas volume increment. Following the nucleation of pores, several pores grow larger than the others, and then they will continue growing and grow faster than the other pores. It can be found that the hydrogen concentration in the remaining liquid as shown in Fig. 4(f) is around 0.45 mL/100 g Al, which is even lower than the initial hydrogen concentration of 0.5 mL/100 g Al. The lower levels of hydrogen can be explained by porosity growth. As the local hydrogen saturation decreases, the pores continue to act as sinks and absorb hydrogen atoms, causing a decrease in the hydrogen concentration of the remaining liquid.

**Figure 5:**
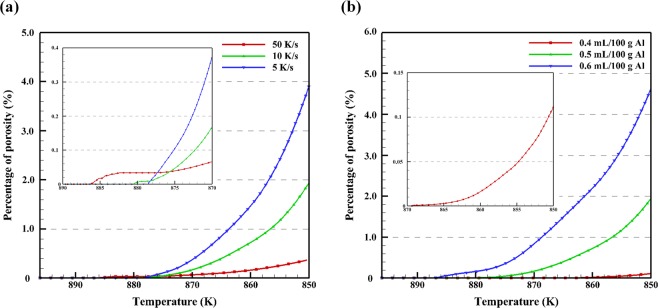
Percentage of porosity vs. temperature with (**a**) different cooling rates of 50 K/s, 10 K/s, and 5 K/s and initial hydrogen concentration of 0.5 mL/100 g Al; and (**b**) with different initial hydrogen concentrations of 0.4, 0.5, 0.6 mL/100 g Al at 10 K/s cooling rate.

Figure 5(a) plots the percentage of porosity vs. temperature with different cooling rates shown in Fig. 4. It can be seen that each of the percentage of porosity curves have an incubation time for the nucleation of pores and for the growth of pores as analyzed above. With the decrease of cooling rate, the final percentage of porosity at the eutectic temperature increases. This is due to that the higher cooling rate limits the time for the nucleated pores to grow. In the higher cooling rate situation where hydrogen atoms contribute less in the growth of pores, they remain in the liquid and lead to the higher hydrogen concentration. On the other hand, with a lower cooling rate, pores begin to nucleate at a lower temperature, and the nucleation period is shorter. This occurs because with a lower cooling rate, the hydrogen accumulated at the S/L interface will have more time to diffuse away at a slightly elevated temperature. It takes more time for the local hydrogen concentration to increase to a value larger than the local hydrogen saturation, and thus pores start to nucleate at a lower temperature. Furthermore, at a lower cooling rate, the hydrogen concentration in almost all of the remaining liquid is higher than the hydrogen saturation before the pore begins to nucleate, which means it will take less time for the hydrogen concentration to increase beyond the hydrogen saturation again after the nucleation of pores. Figure 5(b) shows the porosity percentage vs. temperature with different initial hydrogen concentrations of 0.4, 0.5, and 0.6 mL/100 g Al. It is evident that the final porosity percentage at the eutectic temperature increases with the increase of initial hydrogen concentration. With a lower initial hydrogen concentration, pores start to nucleate at a lower temperature. This is explained by hydrogen diffusion, as with less hydrogen it is able to diffuse away from the S/L interface relatively quickly and maintain a hydrogen concentration below the critical value. When the initial hydrogen concentration is increased, even though hydrogen is diffusing away from the interface, some hydrogen remains and accumulates to a value which satisfies the critical hydrogen concentration point for nucleation. Thus, higher levels hydrogen will nucleate quicker and at higher temperatures when all other conditions held constant. All percentages of porosity resulting from different initial hydrogen concentrations continue to increase as the temperature decreases, even at the end of the solidification. The continued rise in porosity is due to the growth of the pores and the decrease of temperature. This phenomenon leads to the observation that the hydrogen concentration is always higher than the hydrogen saturation, and the hydrogen atoms absorbed by the pores continue contributing to the growth of porosity even following solidification. With the decrease of initial hydrogen concentration, both the total number of pores and the size of pores will decreases. Regarding the hydrogen concentration field, the final hydrogen concentration in the melt is lower when the initial hydrogen concentration is higher. The reason is that the higher initial hydrogen concentration leads to a larger pore radius, which results in a lower local hydrogen saturation, and thus a lower final hydrogen concentration in the remaining melt.

**Figure 6:**
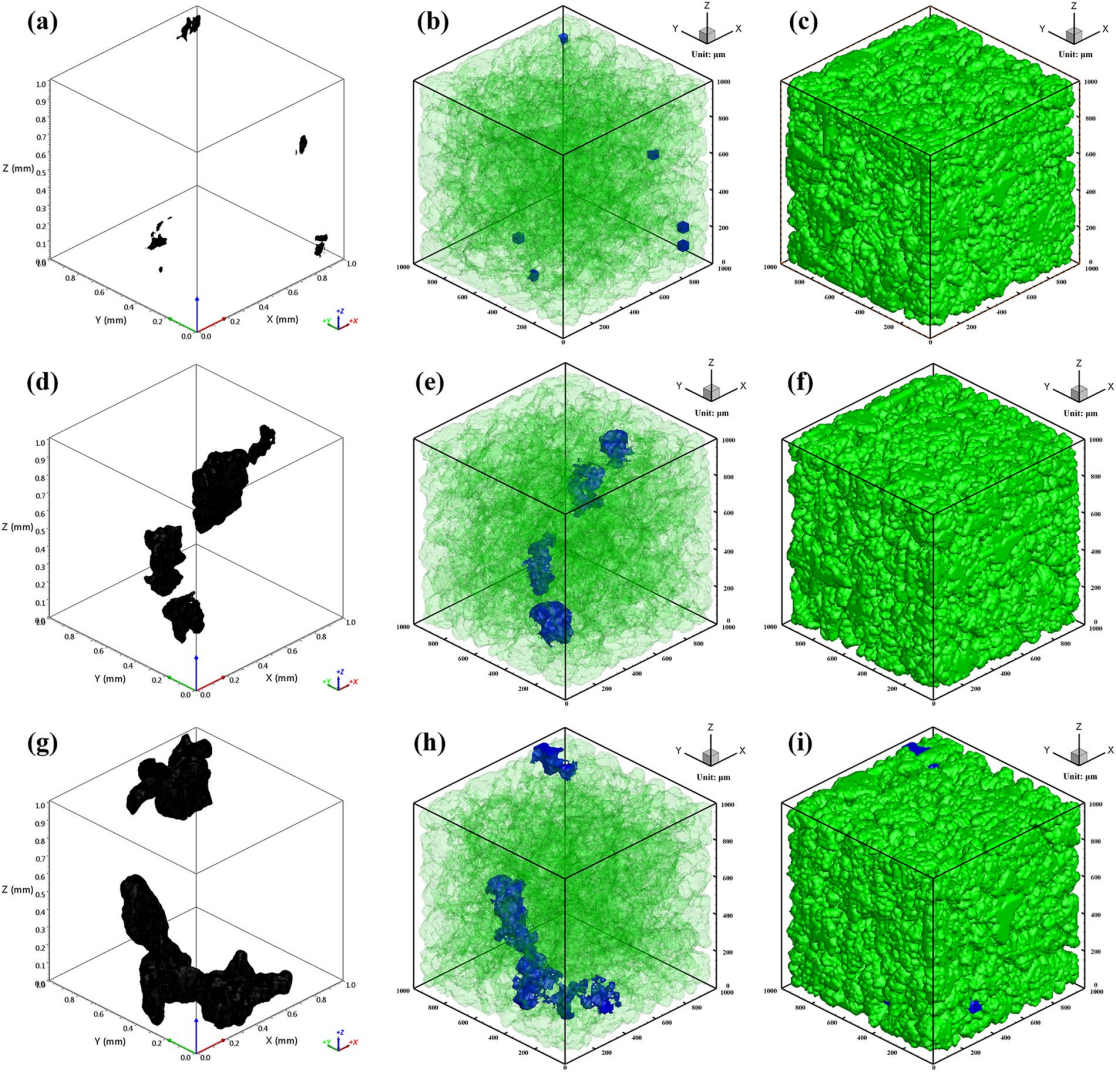
Comparison of the porosity morphology between X-ray microCT results and CA simulated results with different cooling rates: X-ray microCT results (**a,d,g**); porosity morphology in simulated results (**b,e,h**); dendrite morphology (**c,f,i**). The cooling rates are: (**a–c**) 64.8 K/s; (**d–f**) 10.7 K/s; (**g–i**) 2.5 K/s. (Grains are shown in green; porosity is shown in black in (**a,d,g**), and in blue in (**b,e,h**)).

Porosity in casting products can be reduced by controlling the gas quantity in the melt (such as degassing), or optimizing the solidification conditions (such as increasing cooling rates). Since cooling rates vary considerably at different locations of an individual casting due to varying section thickness/cooling lines etc., it will undoubtedly result in varying porosity size, morphology, and formation within the casting. Such a porosity distribution will lead to location-specific mechanical properties which are often unaccounted for in casting design and manufacturing. Figure 6 shows the comparison of the porosity morphology between X-ray micro computed tomography (microCT) measurements and CA simulated results. As shown in X-ray microCT results, with the highest cooling rate in Fig. 6(a), the pores are constrained between the dendrites. The growth of porosity is blocked by the dendrite morphology, and vice versa. With the decrease of cooling rate, the porosity appears to have a larger size and a more rounded morphology. Porosity with transparent dendrites at the end of solidification can be clearly observed in Fig. 6(b,e,h). The pores appear to be located between dendrites. As the cooling rate decreased, the porosity size increased as expected, while the total number of individual pores decreases. This result is in good agreement with the analyses of Atwood *et al*.^3^. Since the pores surrounded by dendrites are not able to escape from the remaining liquid, they form porosity in the final casting. It shows that CA simulation can be used to visualize the porosity morphology by simultaneously simulating both porosity and dendrites, and the simulated results agree well with the experimental results.

**Figure 7:**
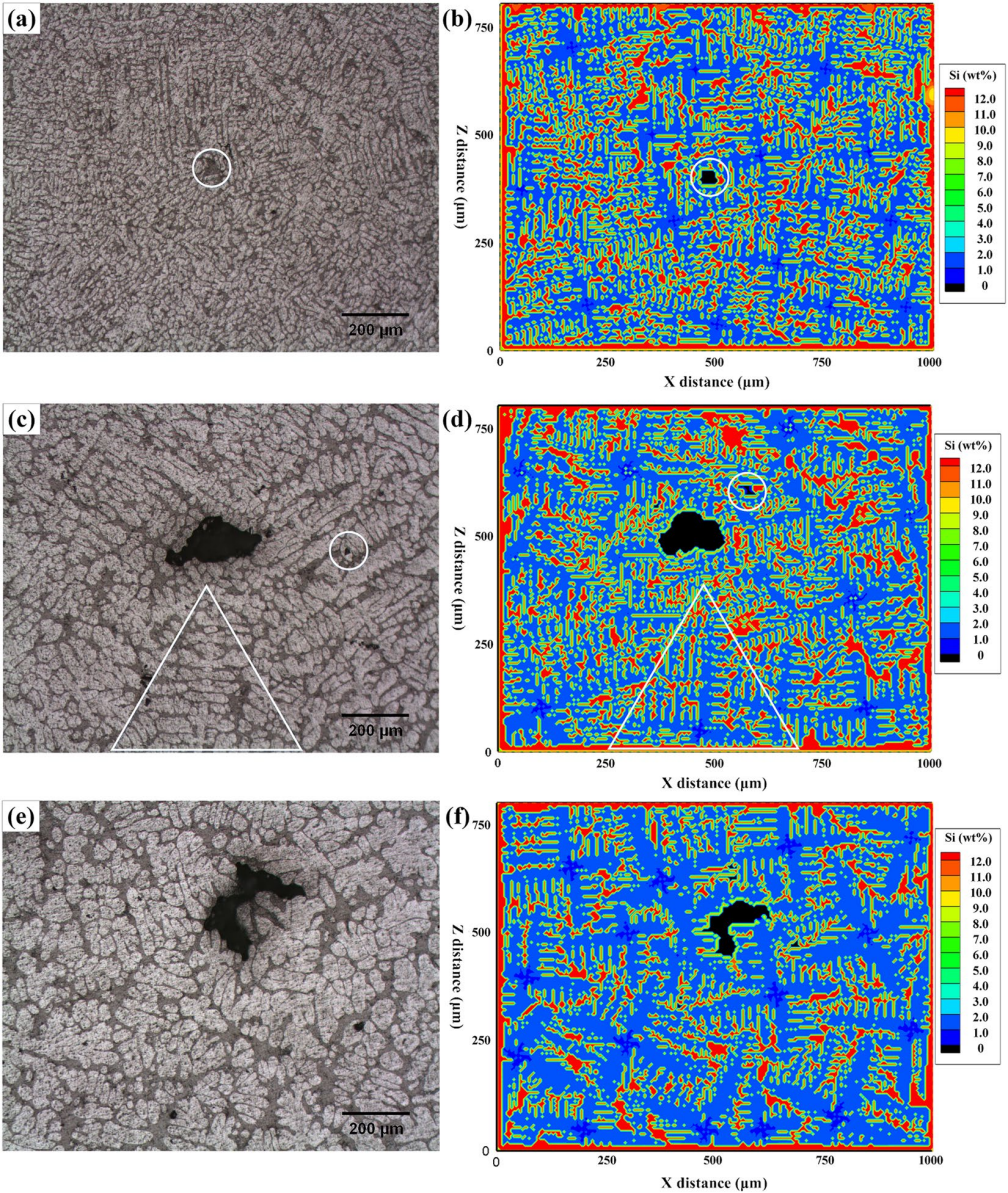
Comparison of 2-D optical microstructure and CA simulated results. Optical metallography results (**a**) 64.8 K/s, (**c**) 10.7 K/s, and (**e**) 2.5 K/s; 2-D sections of CA simulated results (**b**) 64.8 K/s, (**d**) 10.7 K/s, and (**f**) 2.5 K/s.

In the optical microstructure, both dendrites and porosity can be clearly observed. Several pores were identified and examined as shown in Fig. 7. It also can be seen that the dendrite morphologies in the simulated results match well with the experiment results as shown in the white triangle of Fig. 7(c,d). With decreasing cooling rate, the dendrites coarsen and are associated with larger pores. However, the pores did not appear as round morphologies. This is attributed to the 2-D cross-section can only show a slice of complete 3-D information^30^. Nevertheless, it shows that the 2-D simulated results present a good agreement with the optical microstructures, both the dendrite morphology and porosity morphology.

It is clear that the cooling rate affects the final percentage of porosity as analyzed above. However, it should be noted that the porosity size is not uniform at a slow cooling rate. Several pores grow to have large sizes, while others are not able to grow as large as seen in Fig. 4(c). With a larger radius, the local hydrogen saturation is lowered in the melt as discussed above, and results in the pore absorbing more hydrogen and growing to a larger size. Meanwhile, pores with small radius are not able to grow. Smaller pores have higher local hydrogen saturations, and the hydrogen atoms in the liquid melt are absorbed by the larger pores when there is enough time for hydrogen diffusion at a slow cooling rate. Accordingly, the competitive growth among the nucleated pores becomes more obvious with the decrease in cooling rate, resulting in an uneven distribution of pore radius (Fig. 4(c)) and an increased maximum porosity radius (Fig. 7).

**Figure 8:**
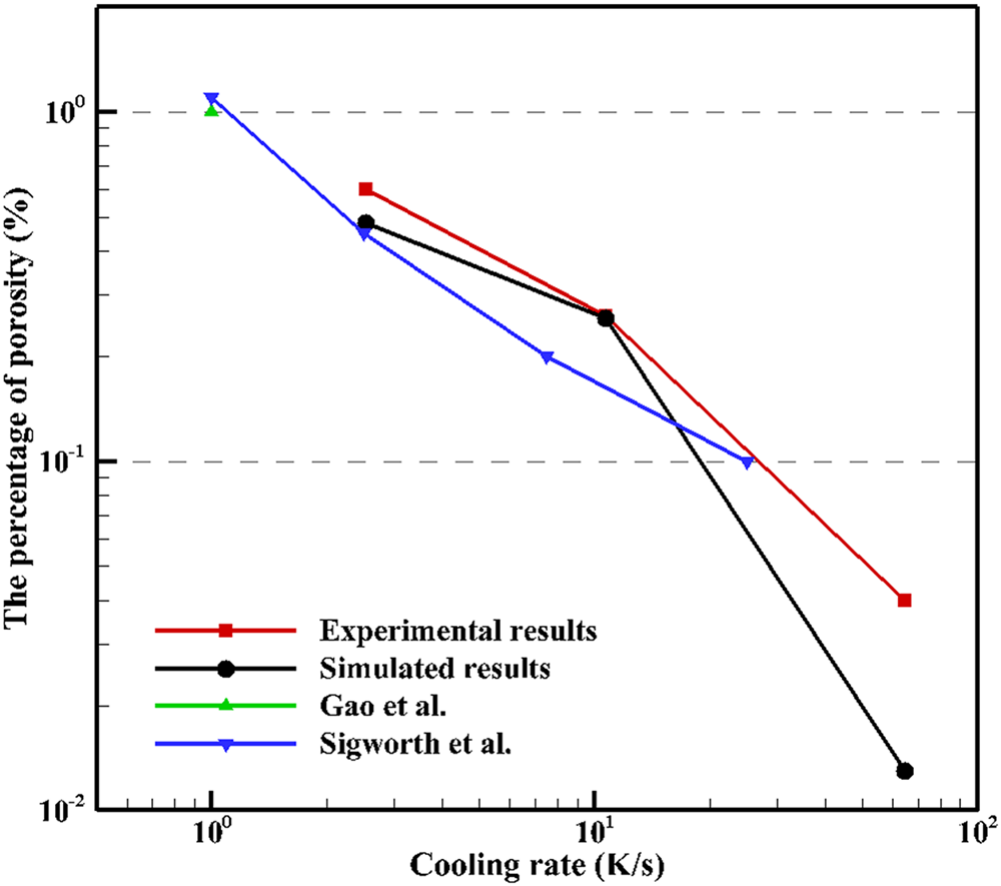
Porosity percentage vs. cooling rate with different cooling rates, and comparison with literatures^26,31^.

Similar phenomenon was noticed by Karagadde *et al*.^23^ who built a model to simulate the hydrogen bubble evolution in micro-scale domain, and studied the effect of cooling rate on average pore radius. They reported that, with increasing cooling rate, the average final pore radius decreases. In the present study the pore radius varied greatly in both the experimental and simulated results. Figure 8 clearly shows that increasing cooling rate decreases the percentage of porosity present within the casting. Both the simulation and experimental results in this study were compared to the experimental results collected by Gao *et al*.^26^ and Sigworth *et al*.^31^, with excellent agreement. Gao *et al*.^26^ calculated the percent porosity of Al-4.5wt.%Cu alloy with 0.3 mL/100 g Al and a cooling rate of 1.0 K/s by building a theoretical model, where the green triangle in Fig. 8 shows the porosity percentage is 1%. Sigworth *et al*.^31^ studied the mechanisms of porosity formation during solidification, and showed the values of the porosity percentage during solidification of A356 alloy with 0.3 mL/100 g Al, which used the same material and initial hydrogen content as in this study. It should be pointed out that the porosity percentage from the simulation is slightly lower than the experimental results. This discrepancy can also be found in porosity morphology shown in Figs 6 and 7, which is attributed to micro-shrinkage. Micro-shrinkage occurs at the end of solidification due to the lack of liquid feeding and increases the total percentage of final porosity. It should also be noted that several assumptions are adopted in the present modeling and simulation which is different from the real non-equilibrium solidification. The solidification process is assumed to be equilibrium, and only hydrogen gas porosity is considered in the present model. As shown in Fig. 3, approximately 48% of the melt has not solidified at the end of simulation, which is supposed to solidify as eutectic. Further eutectic solidification is not considered in the model since the formation of gas porosity has mostly completed before that. Although shrinkage porosity is not considered in the present porosity model, the evolution of hydrogen porosity (which is dominant in most solidification products of aluminum alloys) can be predicted, and the morphology and distribution of hydrogen porosity can be quantitatively obtained. Validating the present model offers an excellent opportunity to predict location-specific microstructure and mechanical properties of final castings. By understanding where and how pores will evolve in a cast structure, it will help optimize the design of castings and reduce scrap rates in casting production. Therefore, the validated porosity model will provide an indispensable part from location-specific microstructure to location-specific mechanical properties in ICME design and manufacturing of lightweight Al castings.

In summary, a 3-D CA model was built to simulate the formation and evolution of hydrogen porosity coupled with grain growth during solidification process of Al-7wt.%Si-0.3wt.%Mg ternary alloy. The concurrent nucleation and growth of both grains and pores were examined, and the redistribution and diffusion of solute and hydrogen were obtained using the present model. By applying the 3-D CA model, multi-grain growth with porosity evolution can be simulated. From simulated results, porosity morphology and distribution can be visualized qualitatively, and porosity size and percentage can be computed quantitatively. It is found that, with lower cooling rates, the final porosity percentage and the maximum porosity size increases, while the total number of individual pores decreases. In addition, for higher cooling rates, porosity nucleates at higher temperatures and starts to grow at lower temperatures. With higher initial hydrogen concentrations, the final percentage of porosity increases, and both the number and the size of porosity also increase. In addition, wedge die casting experiments were conducted to generate samples with different cooling rates, and X-ray microCT measurements were performed on the samples to obtain quantitative porosity information. The percentage and the distribution of porosity of simulated results agree well with the experimental results. This model can serve as an indispensable part in connecting location-specific process conditions and location-specific microstructure models for ICME of cast products.

## Methods

A 3-D cellular automaton model is developed to investigate the nucleation and growth of both hydrogen gas pores and grains. By applying the model, the nucleation and growth of hydrogen gas pores and grains during solidification of ternary aluminum alloys can be simulated in 3-D. Additionally, the solute and hydrogen redistribution can be calculated and the size, percentage and distribution of porosity can be obtained. Further, the effect of cooling rate and initial hydrogen concentration on the porosity size and morphology can be evaluated. In CA modeling, a discrete number of cells are given states including: (1) gas cell (*f*_*G*_ = 1), (2) solid cell (*f*_*S*_ = 1), (3) liquid cell (*f*_*G*_ + *f*_*S*_ = 0), (4) the solid/liquid (S/L) interface cell (0 < *f*_*S*_ < 1, and *f*_*G*_ = 0), (5) the gas/liquid (G/L) interface cell (0 < *f*_*G*_ < 1, and *f*_*S*_ = 0), (6) the gas/solid (G/S) interface cell (*f*_*G*_ + *f*_*S*_ = 1), and (7) the gas/liquid/solid (G/L/S) interface cell (0 < *f*_*G*_ + *f*_*S*_ < 1), where *f*_*G*_ and *f*_*S*_ are the gas fraction and solid fraction of the cell, respectively. The state of each cell is then updated or transformed based on a set of neighborhood conditions as the solidification process continues. More detailed explanation of the CA process can be found in the authors’ previous publications^32,33^. Assumptions adopted in this paper include: (1) only hydrogen pores are considered; (2) the effect of fluid flow is not considered; (3) shrinkage porosity due to solidification shrinkage and inadequate feeding during solidification is ignored; and (4) the eutectic solidification is not considered. As solidification proceeds, grains begin to nucleate and grow. Solute (Si and Mg) is rejected from the solid, and accumulates at the S/L interface due to the phase equilibrium. Since hydrogen is far less soluble in solid Al than in liquid Al, the excess atomic hydrogen is rejected from the solid into the remaining liquid phase. With the increase of hydrogen concentration in the liquid, molecular hydrogen pores form in the gaseous state when the atomic hydrogen reaches the solubility limit in the liquid. Based on the local hydrogen concentration levels and the diffusion rate, pores are able to grow or shrink. As the temperature decreases, thermodynamically stable grains and the hydrogen pores will grow. Firstly, the nucleation and the growth of solid grains is primarily controlled by solute and temperature distributions. The continuous nucleation distribution proposed by Rappaz^34,35^ was used. Due to the fact that the rejected solute during solidification increases the solute concentration^36^, the liquidus temperature decreases since the equilibrium partition coefficient is lower than 1, which leads to a decrease in local undercooling^32,37–39^. The local undercooling was adopted to accurately describe the location specific nucleation phenomena. Local equilibrium at the S/L interface and solute diffusion can be expressed as: ${C}_{i}^{S\ast }={k}_{i}{C}_{i}^{L\ast }$, $\frac{\partial {C}_{i}^{E}}{\partial t}=\nabla \cdot \sum _{j=Si,Mg}{D}_{ij}\nabla {C}_{i}^{E}+({C}_{i}^{L\ast }-{C}_{i}^{S\ast })\frac{\partial {f}_{s}}{\partial t}$, where *k*_*i*_ is the solute partition coefficient of the solute element *i*, ${C}_{i}^{S\ast }$ and ${C}_{i}^{L\ast }$ are the solute compositions at the S/L interface in solid and liquid respectively, ${C}_{i}^{E}$ is the solute concentrations in phase *E* (superscript *E* represents *S* in solid or *L* in liquid), and *D* is the diffusion coefficient^33^. Based on the lever rule of both solute elements Si and Mg, the solid fraction and the solid fraction increment can be calculated^39,40^.

Secondly, gaseous pore nucleation only occurs when the gas dissolves in the liquid (${C}_{H}^{L}$) and exceeds the critical supersaturation^27^. The hydrogen saturation in melt Al-Si-Mg can be described based on Sievert’s law as: ${S}_{H}^{L}=\sqrt{\frac{{P}_{G}}{{P}_{0}}}{10}^{(-\frac{2760}{T}+2.796-0.0119{C}_{Si}^{L}+0.017{C}_{Mg}^{L})}$ (mL/100 g Al)^3,18^, where ${P}_{G}={P}_{0}+2\gamma /{r}_{P}$ is the internal pressure of a gas pore, *P*_0_ is the standard atmospheric pressure, γ is the surface tension of the G/L interface, *r*_*p*_ is the pore radius, and ${C}_{Si}^{L}$ and ${C}_{Mg}^{L}$ are the solute concentrations of Si and Mg in melt Al, respectively. The supersaturated hydrogen originates from liquid-solid transformation^40,41^. Furthermore, any hydrogen that is located within the liquid state is able to be absorbed by adjacent pores (as shown in Fig. 1(c)). The diffusion of hydrogen can be expressed as: $\frac{\partial {C}_{H}^{E}}{\partial t}=\nabla \cdot ({D}_{H}^{E}\nabla {C}_{H}^{E})+{C}_{H}^{E}(1-{k}_{H})\frac{\partial {f}_{S}}{\partial t}-(1-{f}_{S}-{f}_{G})({C}_{H}^{L}-{S}_{H}^{L})$, where *C* and D are the hydrogen concentrations and the diffusion coefficient respectively, and *k*_*H*_ is the partition coefficient of hydrogen^40,41^. Then the gas volume increment of the pore, $\Delta V$, can be related to the hydrogen absorbed at the interface cell, *V*_*G*_, in one time step interval based on the ideal gas law: ${P}_{G}\Delta V={N}_{G}RT={P}_{0}{V}_{G}$, ${N}_{G}=\sum _{A}(1-{f}_{S}-{f}_{G}){V}_{cell}\rho ({C}_{H}^{L}-{S}_{H}^{L})\frac{{P}_{0}}{RT}$, $\Delta V=\frac{{P}_{0}}{{P}_{G}}{V}_{G}=\frac{{P}_{0}}{{P}_{G}}\sum _{A}(1-{f}_{S}-{f}_{G}){V}_{cell}\rho ({C}_{H}^{L}-{S}_{H}^{L})$, where *N*_*G*_ means the amount of hydrogen, *R* is the gas constant, *Σ* includes all G/L interface cells of the pore, *V*_*cell*_ is the volume of a cell, and *ρ* is the density of liquid Al melt. The volume change of the G/L interface cell (*i, j, k*) of the pore A at any given increment can be evaluated as: $\Delta V(i,j,k)=\Delta V\frac{{G}_{G}(i,j,k)}{{\sum }_{A}{G}_{G}}$, where ${G}_{G}(i,j,k)$ is geometrical factor of the G/L interface cell (*i, j, k*). The geometrical factor *G*_*G*_ for 2-D system proposed by Zhu *et al*.^27^ is extended to 3-D system as follows: ${G}_{G}(i,j,k)=min[1,\frac{1}{3}(\mathop{\sum }\limits_{m=1}^{6}{S}_{m}^{I}(i,j,k)+\frac{1}{\sqrt{2}}\mathop{\sum }\limits_{m=1}^{12}{S}_{m}^{II}(i,j,k)+\frac{1}{\sqrt{3}}\mathop{\sum }\limits_{m=1}^{8}{S}_{m}^{III}(i,j,k))]$, $${S}^{I}(i,j,k),{S}^{II}(i,j,k),{S}^{III}(i,j,k)=\{\begin{array}{c}0,({f}_{G}(i,j,k) < 1)\\ 1,({f}_{G}(i,j,k)=1)\end{array}$$, where *S*^*I*^, *S*^*II*^ and *S*^*III*^ are the state of the nearest 6 neighbor cells, the second-nearest 12 neighbor cells, and the third-nearest 8 neighbor cells, respectively.

The calculated time step is based on the Courant–Friedrichs–Lewy criterion for the stability of explicit solute diffusion, and is expressed as: $\Delta {t}_{C}=\frac{1}{5}min(\frac{\Delta {x}^{2}}{|{D}_{ij}^{L}|},\frac{1}{\Delta {f}_{s,max}})$. The time step for hydrogen diffusion, $\Delta {t}_{H}$, is determined by: $\Delta {t}_{H}=\frac{1}{5}\frac{\Delta {x}^{2}}{{D}_{H}^{L}}$, where ${D}_{H}^{L}$ is the diffusion coefficient of hydrogen in melt Al. Due to the fact that the diffusion coefficient of hydrogen in the melt Al is much larger than that of solute (Si or Mg), the time step used in hydrogen diffusion is much smaller than that of solute diffusion. To increase the computational efficiency, two different time steps are used in the simulations. The diffusion of hydrogen is calculated for ${\rm{Nt}}=\Delta {t}_{C}/\Delta {t}_{H}$ times using $\Delta {t}_{H}$. $\Delta {t}_{C}$ is used in the calculation of the diffusion of solute Si, and the evolution of grains and gas pores. In this CA model, the anisotropy^36,42–45^ from the regular coordinate system in a 3-D system was employed as detailed in previous research^46^. Based on the definitions above, porosity evolution is coupled with grain growth. Ternary Al-7wt.%Si-0.3wt.%Mg was used as an approximate composition in the above modeling and simulations. The material parameters in the literatures^14,27,39,47^ were adopted in the simulation.

To validate the CA model, a V-shaped wedge casting experiment was performed. Primary A356 ingots were melted at 973 K (700 °C) in graphite crucibles coated in boron nitride using an electric resistance furnace, and the surface dross was removed prior to casting. K-type thermocouples with 0.2 mm diameter were placed in three different heights along the wedge casting, corresponding to different thickness, i.e. different cooling rates. Thermocouple wires were shielded in alumina tubes, while the thermocouple junctions were exposed directly to the melt for faster response and improved accuracy. The cooling curves were collected using a computer data acquisition system (NI 9219 from National Instruments) with a sampling rate of 50 readings per second. The instantaneous cooling rates at different locations calculated directly at the liquid temperature were 64.8, 10.7, and 2.5 K/s, respectively.

Prior to casting, the initial hydrogen concentration was measured using the Straube-Pfeiffer vacuum solidification test^48^ or equivalently the reduced pressure test (RPT)^49^. In this study, the RPT was carried out on three specimens, followed by density measurements to obtain the average density of the melt. For each test, approximately 100 g of A356 alloy was first melted in a muffle furnace at 973 K (700 °C). Then the sample melt was poured into a steel cup and solidified at a pressure of 100 mmHg for 20 minutes. The average density was calculated based on the Archimedes Principle after the dry weight and wet weight of each sample was measured. The hydrogen content can be obtained as ${C}_{H}^{0}$ = 0.3 mL/100 g Al. After wedge casting experiments, X-ray microCT measurements were performed to characterize the porosity in the casting samples.

## Supplementary information

3-D rotation video of Fig. 6(h)

3-D rotation video of Fig. 6(g)

## Data Availability

The data that support the findings of this study are available from the corresponding author upon reasonable request.
